# *Anacyclus pyrethrum* enhances fertility in cadmium-intoxicated male rats by improving sperm functions

**DOI:** 10.1186/s12906-024-04711-y

**Published:** 2024-11-27

**Authors:** Aya A. Mahmoud, Mennat Allah M. Shaaban, Wesam T. Basal

**Affiliations:** Department of Zoology, Faculty of Science, Cairo University, Giza, 12613 Egypt

**Keywords:** *Anacyclus pyrethrum*, Apoptosis, Cadmium toxicity, Oxidative stress, Sex hormone receptors, Sperm DNA damage

## Abstract

**Background:**

Environmental pollutants, particularly heavy metals, have been frequently connected to male infertility. Cadmium was previously shown to reduce male fertility by causing oxidative stress. *Anacyclus pyrethrum* is a well-known medicinal plant. Most of its parts, notably the roots, have excellent antioxidant and anti-inflammatory properties. The present study investigated the potential ability of *Anacyclus pyrethrum* to protect male rats against cadmium reproductive toxicity.

**Methods:**

Twenty-eight adult Wistar male rats (8 weeks old) weighing (170-200g) were randomly divided into four groups (*n* = 7): group (1) the control, group (2) was orally administrated with *Anacyclus pyrethrum* extract (100mg/kg) for 56 consecutive days, group (3) received a single intraperitoneal (IP) injection of cadmium chloride (1mg/kg), and group (4) received a single IP dose of CdCl_2_ followed by 8 weeks of oral *Anacyclus* extract treatment.

**Results:**

Cadmium **Cd** toxicity resulted in a significant decrease in the concentration of antioxidant enzymes (superoxide dismutase **SOD** and glutathione peroxidase **GPx**) in the semen coupled with a significant rise in malondialdehyde **MDA** level. Consequently, sperm analysis parameters were significantly affected showing decreased motility, viability, concentration and increased morphological aberrations. DNA fragmentation was also detected in the sperms of rats exposed to Cd using comet assay. Serum levels of testosterone **T**, follicle stimulating hormone **FSH**, and luteinizing hormone **LH** were significantly decreased. The mRNA expression levels of sex hormone receptors (***FSHR***, ***LHR*** and ***AR***) in the testis of the Cd exposed rats were significantly decreased. Expression levels of *Bax* and *Bcl2* genes in the sperms of Cd intoxicated rats were also affected shifting the *Bax*/*Bcl2* ratio towards the induction of apoptosis. Co-treatment with the *Anacyclus pyrethrum* extract restored the oxidative enzymes activities and decreased the formation of lipid peroxidation byproduct, which in turn ameliorated the effect of Cd on sperm parameters, sperm DNA damage, circulating hormone levels, gene expression and apoptosis. These results indicate that *Anacyclus pyrethrum* could serve as a protective agent against cadmium-induced sperm toxicity.

**Conclusion:**

Taken together, it can be concluded that the antioxidant activities of *Anacyclus pyrethrum* restored the semen quality and enhanced fertility in Cd-intoxicated male rats.

**Supplementary Information:**

The online version contains supplementary material available at 10.1186/s12906-024-04711-y.

## Background

Exposure to environmental pollutants, including heavy metals, has been repeatedly linked to male infertility [1]. The data collected from human epidemiological studies and research work on rodents proved that Cadmium (Cd) exposure affects male fertility by inducing oxidative stress, compromising DNA integrity, decreasing sperm motility and viability, sabotaging the endocrine function, impairing spermatogenesis, and modulating mRNA expression of responsible genes [2–5]. A significant increase in the number of abnormal sperm, with a marked decrease in the viability, motility, concentration, and fertilization capacity via Cd-mediated oxidative damage has been detected in both in vivo and in vitro studies on human, rat, mouse, and caprine sperm [6, 7]. A complete absence of spermatozoa was also observed by scanning electron microscopy in the testis of rat administered with a low dose of Cd [6]. Defective sperm functions are the most prevalent and difficult to treat cause of male infertility [8].

Among various causes, Cd imposed oxidative stress (OS) has been recognized for affecting the fertility status and physiology of spermatozoa [9]. Uncontrolled production of reactive oxygen species (ROS) that exceeds the antioxidant capacity of the seminal plasma leads to oxidative stress (OS) which is harmful to spermatozoa [10]. Unfortunately, the evaluation of OS is not a routine clinical practice yet. Evaluation of OS status should be validated and simplified to be routinely performed without the need for sophisticated equipment [11]. For assessment of oxidative stress in semen, certain markers are analyzed spectrophotometrically including the levels of malondialdehyde (MDA) and semen concentration of glutathione peroxidase (GPx) and superoxide dismutase (SOD) [12, 13]. Previous work has pointed out the preferential binding of Cd to the sulfhydryl groups of certain antioxidant enzymes like SOD and GSH. The depletion of the ─SH group is considered as an indirect mechanism of Cd-induced oxidative stress [6, 14]. In the routine semen analysis sperm concentration, motility, and morphology are analyzed. However, 15% of males with normal sperm analysis profiles still showed fertility problems. Therefore, to duly diagnose male infertility, it may be necessary to measure other sperm parameters [15–17]. Increased OS levels result in DNA oxidation and compromises its integrity in the sperms. DNA fragmentation was shown to be a major indicator of fertility potential, even more than conventional semen parameters but its inclusion in routine semen analysis is still undervalued [17]. Comet assay for sperms has been applied in several studies to evaluate the effect of reproductive toxins and genotoxins in male human and rats [18, 19].

Serum levels of FSH, LH and T are good indicators of fertility status [20, 21]. Cd is a known endocrine disruptor that mainly targets hypothalamic-pituitary axis, hence may affect the circulating levels of sex hormones [22]. Low serum levels of these hormones were previously discovered in male rats exposed to cadmium [23, 24].

Several studies provided solid evidence for the pivotal role of oxidative stress in the negative regulation of steroidogenesis and spermatogenesis by reduction of mRNA levels produced by the responsible genes [25]. Oxidants, antioxidants, and other determinants of the intracellular reduction–oxidation state play an important role in the regulation of gene expression [26]. Spermatogenic cells eliminate oxidative DNA by apoptosis through p53-dependent and -independent mechanisms, which at higher activities may lead to male infertility [27]. Realtime PCR, a commonly accepted tool now in reproductive biology, is used for measuring the expression levels of genes involved in production of proteins affecting spermatogenesis and the induction of apoptosis in germinal cells of testis and spermatozoa [28, 29].

*Anacyclus pyrethrum*, also known as Akarkara, is an important annual medicinal herb. Most of its parts, especially roots, are used in traditional medicine [30]. It was reported earlier that the roots of *Anacyclus pyrethrum* possess some powerful pharmacological properties including anticancer, memory-enhancing, immunostimulant, antidepressant, anticonvulsive, aphrodisiac, androgenic, antibacterial, antioxidant, and anti-inflammatory effects [31–33]. It is also well-known in Indian medicine as a tonic and rejuvenator [34, 35].

The therapeutic ability of *Anacyclus pyrethrum* may be attributed to its antioxidant effect which in turn prevents DNA oxidative damage and cytotoxicity [32, 33, 36]. Its antioxidant potential has been correlated with its uses as an anticonvulsant, brain tonic [37], anti-inflammatory [38], anti-cancer [39], protective agent against neurological disorders associated with oxidative stress (such as Alzheimer’s and Parkinson’s diseases) [40] and anti-diabetic [41]. Recently, Baslam et al. proved that *Anacyclus pyrethrum* extract protects male rats' brain tissue from oxidative damage by reducing MDA levels and increasing CAT and SOD [42, 43].

The antioxidant and anti-inflammatory attributes of *Anacyclus pyrethrum* were previously employed for the systemic detoxification of copper induced toxicity in zebrafish [44]. Most importantly, the root extract of *Anacyclus pyrethrum* was shown to improve fertility via four different pathways: 1. direct or indirect influence on serum levels of sex hormones [35, 45]. 2. Enhancing spermatogenesis which ultimately helps in improving reproductive potential [34, 45]. 3. Reduction of sperm morphological abnormalities by the antioxidant effects of *Anacyclus* extract [46]. 4. Protection against sexual disorders induced by oxidative stress and apoptosis [47]. Previous studies showed that administration of *Anacyclus* root extract increased fertility of male rats by elevating sperm count, motility, vitality, and seminal fructose level. It was also found to increase the serum levels of testosterone, luteinizing hormone and follicle stimulating hormone in healthy male rats [34, 45–50]. Moreover, Sharma et al. observed sustained sexual function improvement in male rats 7 and 15 days after withdrawal of *Anacyclus* extract compared to testosterone-treated rats [45].

Historically, animal models played a pivotal role from early reproductive basic research to modern techniques like artificial insemination and in vitro fertilization [51]. The Wistar rat model offers translational relevance to human fertility owing to similarities in reproductive anatomy, hormonal regulation, and physiological responses to human [52].

This study aims at the evaluation of Cd-induced sperm toxicity by measuring the effect of oxidative stress on semen analysis parameters, DNA fragmentation, serum hormonal levels, and the expression levels of spermatogenic and apoptotic genes. It also investigated the ameliorative role of the medicinal herb *Anacyclus pyrethrum* in regaining semen quality.

## Materials and methods

### Materials

Cadmium chloride (CdCl_2_) was purchased from Sigma-Aldrich (St. Louis, MO, USA). High quality *Anacyclus pyrethrum* roots, commonly known as Akarkara, was purchased from MB herbals (Ahmedabad, India).

### Preparation and identification of Anacyclus* pyrethrum* extract

The biologically active components were extracted from 100 g of well grinded dried *Anacyclus pyrethrum* roots using methanol at room temperature, following the methodology described by Manouze et al. [53]. The filtrate was evaporated by a rotary evaporator (Hei-Vap Value HL/HB/G1, Heidolph, Schwabach, Germany) at 30°C. Stock solution (100 mg/mL) was prepared by dissolving the residues (16.8% w/w) in distilled water with continuous stirring for 12 h. The solution was stored at 4℃ and used to prepare 20 mg/mL working solution.

The extract's phenol content was determined using High Performance Liquid Chromatography (HPLC) (Agilent 1260 infinity, Waldbronn, Germany). All experiments and techniques adhere to the IUCN Policy Statement on Research Involving Species at Risk of Extinction and the Convention on the Trade in Endangered Species of Wild Fauna and Flora.

### Experimental animals

Twenty-eight adult (8 weeks old) Wistar male rats (weighing 170–200 g) were purchased from the National Research Center, Egypt. The animals were examined by a vet and kept in a climate-controlled room (20–22 °C temperature and 50–55.5% relative humidity) under an alternating 12-h light/dark cycle. The rats were supplied with standard chow and water ad libitum [54]. All experiments and procedures followed ARRIVE guidelines and were approved by The Institutional Animal Care and Use Committee (IACUC), Faculty of Science, Cairo University (approval number (CU/I//F/53/21)). All experiments were carried out consistently with relevant guidelines and regulations.

### Experimental design

Male Wistar rats were randomly and equally divided into four groups (*n* = 7) as follows:

- Group 1 (G1) or the control group, received a single intraperitoneal (IP) injection with CdCl_2_ vehicle (saline) and 8 weeks oral gavage of water (vehicle of *Anacyclus pyrethrum* extract).
- Group 2 (G2) received 100 mg/kg of the Anacyclus pyrethrum extract daily for 8 weeks using oral tube [35].
- Group 3 (G3) received a single IP injection of 1 mg/kg of CdCl_2_ dissolved in saline solution [55].
- Group 4 (G4) received a single IP injection of 1 mg/kg CdCl_2_, simultaneously with the 8-week Anacyclus extract treatment according to Basal et al. [54].

After eight weeks, animals were euthanized by intraperitoneal injection of 50 mg/kg of sodium pentobarbital followed by decapitation.

### Sperm motility and concentration

Sperms in epididymal suspension were loaded into a hemocytometer's counting chamber and covered with a cover slip. Five fields per sample were examined randomly on a warm plate of CX43 phase-contrast microscope (Olympus, Tokyo, Japan) at 40 × magnification. Sperm motility was determined according to the method given by El-Magd et al. [56] and sperm concentration by Yokoi et al. [57].

### Sperm viability and morphology

Sperm viability and morphological abnormalities were detected using eosin-nigrosin (EN) staining (Sigma-Aldrich, St. Louis, MO, USA). At least 200 spermatozoa from each animal were analyzed using a BX40 bright field microscope (Olympus, Tokyo, Japan) at 400 × magnification. The proportion of sperm cell viability and abnormalities was recorded according to the standards reported by Okamura et al. [58].

### Evaluation of DNA fragmentation using comet assay

The alkaline comet assay, previously described by Tice et al. [59], was used to determine the degree of DNA strand breaks in the sperms of control and treated groups. About 1 g of sperm cell pellet was homogenized in 1 ml of cold mincing solution and then resuspended in darkness. Komet 5.0 analysis system developed by Kinetic Imaging, Ltd. (Liverpool, United Kingdom) connected to a charge-coupled device (CCD) camera and 40 × objective of fluorescence microscope (Olympus, Tokyo, Japan) with excitation filter 420–490 nm (issue 510 nm) was used for comet analysis.

### Assessment of oxidative stress markers

Oxidative stress markers in the semen of cadmium and/or *Anacyclus pyrethrum* treated rats were assessed colorimetrically using commercial kits (Bio-diagnostic, Giza, Egypt). The level of malondialdehyde (MDA) (Cat. No. MD2529) and the activities of superoxide dismutase (SOD) (Cat. No. SD2521), and glutathione peroxidase (GPx) (Cat. No. GP2524) measurements were carried out following the manual provided by the manufacturer and the guidelines described by Ohkawa et al. [60], Nishikimi et al. [61], and Paglia and Valentine [62] respectively.

### Hormonal assay

Serum reproductive hormone levels were measured using commercial ELISA kits for luteinizing hormone (LH) (Novus Biologicals, Cat. No. NBP2-61257, Abingdon, USA), follicle-stimulating hormone (FSH) (Abnova, Cat. No. KA2330, Taipei, Taiwan), and testosterone (Cusabio, Cat. No. CSB-E05097r, Houston, USA). Analysis was conducted at 450 nm according to the manufacturer's protocol [63].

### Determining fold change of hormonal receptors and apoptotic genes using real time PCR

Total RNA was extracted from testis tissue and semen using GeneJET™ RNA extraction kit (Thermo Fisher Scientific Inc., Rochester, New York, USA, #K0731) according to the manufacturer’s protocol. RNA was converted into complementary DNA (cDNA) using Revert Aid H minus First Strand cDNA Synthesis kits (Thermo Fisher Scientific Inc., Rochester, New York, USA, #K1632) according to the manufacturers protocol. The isolated cDNA along with *β-actin* (as an internal reference) were amplified using 2X Maxima SYBR Green/ROX qPCR Master Mix following the manufacturer protocol (Thermo Fisher scientific Inc., Rochester, New York, USA, # K0221) and gene specific primers (Table 1). The polymerase chain reaction mixture was placed in a StepOnePlus real time thermal cycler (Thermo Fisher Scientific Inc., Rochester, New York, USA) to produce a melt curve. The fold changes of target genes were calculated by normalizing the quantities critical threshold (Ct) of target genes with (Ct) of the housekeeping gene (*ß-actin*) using the 2^−∆∆Ct^ method [64].

**Table 1:** Sequence of Forward and reverse primers used in qPCR

Gene	Forward primer (^/^5 ------ ^/^3)	Reverse primer (^/^5 ------ ^/^3)
*FSHR*	GAGTCATCCCGAAAGGATCA	TAAAATGACTGGCCCAGAGG
*LHR*	AGAGTGATTCCCTGGAAAGGA	TCATCCCTTGGAAAGCATTC
*AR*	TTACGAAGTGGGCATGATGA	ATCTTGTCCAGGACTCGGTG
*Bax*	ACACCTGAGCTGACCTTG	AGCCCATGATGGTTCTGATC
*Bcl2*	ATCGCTCTGTGGATGACTGAGTAC	AGAGACAGCCAGGAGAAATCAAAC
*β-actin*	ACTATTGGCAACGAGCGGTT	CAGGATTCCATACCCAAGAAGGA

### Statistical analysis

All data was displayed as means ± S.E. Shapiro–Wilk test was used to check the normality of data distribution. Estimation of statistical significance by one-way analysis of variance (ANOVA) was carried out using SPSS software (SPSS Inc. Released 2009. PASW Statistics for Windows, Version 18.0. Chicago, USA) and individual comparisons were obtained using Duncan's multiple range test (DMRT). P-values less than 0.05 were considered statistically significant.

## Results

### Identification of *Anacyclus pyrethrum* methanolic extract using HPLC

**Fig. 1:**
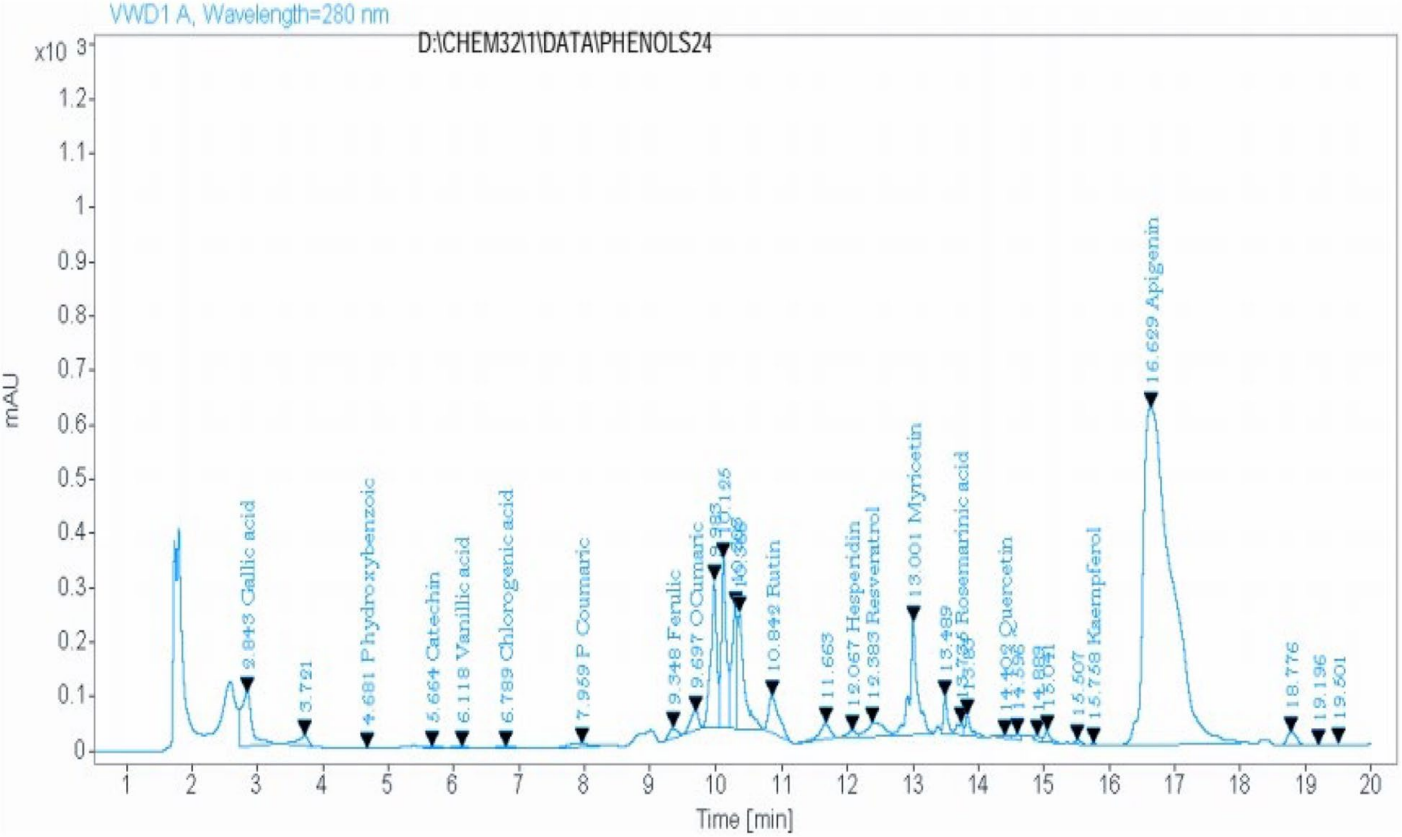
HPLC analysis of the methanolic extract of *Anacyclus pyrethrum* roots

HPLC analysis of the methanolic extract of the *Anacyclus pyrethrum* roots revealed the presence of sixteen phenolic compounds, as shown in Table 2 and Fig. 1.

**Table 2:** HPLC analysis of the methanolic extract of *Anacyclus pyrethrum* roots. Wavelength = 280 nm

Number	Phenolic content	Retention time (min)	Area	Quantity (mg/kg)
1	Gallic acid	2.84	1127.27	111.559
2	P- hydroxybenzoic	4.68	11.51	8.728
3	Catechin	5.66	39.20	56.674
4	Vanillic acid	6.12	33.16	16.388
5	Chlorogenic acid	6.79	28.07	26.099
6	P- coumaric acid	7.96	159.96	35.254
7	Ferulic acid	9.35	155.56	175.342
8	O- coumaric acid	9.70	294.58	51.599
9	Rutin	10.84	775.87	322.473
10	Hesperidin	12.07	106.79	64.190
11	Resveratrol	12.38	435.11	375.787
12	Myricetin	13.00	1701.63	604.420
13	Rosemarinic acid	13.74	161.08	203.625
14	Quercetin	14.40	71.80	227.703
15	Kaempferol	15.76	12.33	5.129
16	Apigenin	16.63	18442.21	822.841

### Semen analysis

#### Sperm motility and concentration

**Fig. 2:**
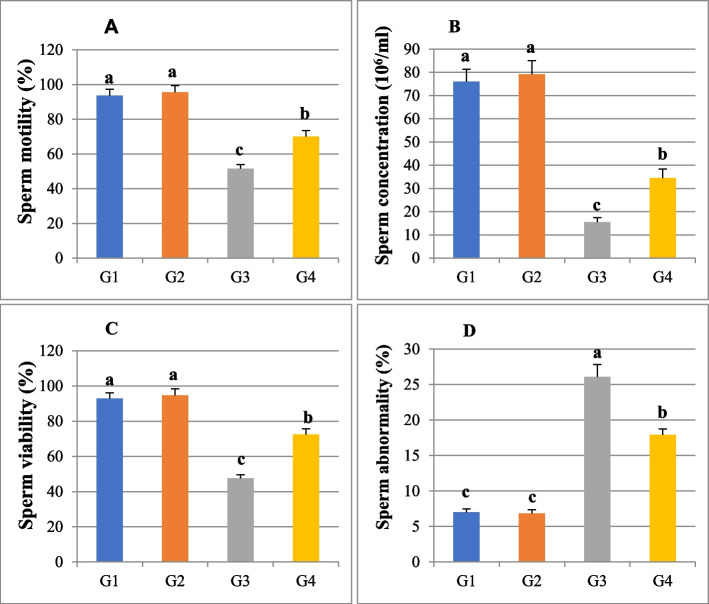
A histogram showing the effect of cadmium or/and *Anacyclus pyrethrum* extract treatment for eight weeks on sperm parameters of male Wistar rats in all experimental groups. **A** Sperm motility (%), (**B**) Sperm concentration (10^6^/ml), (**C**) Sperm viability (%), and (**D**) Sperm abnormality (%). G1: Control, G2: *Anacyclus* extract treated group, G3: Cadmium treated group, G4: Cadmium and *Anacyclus* extract treated group. Data is presented as mean ± standard error (S.E.). Different letters represent significant differences (*P* ≤ 0.05) between experimental groups

Sperm analysis of the rats' epididymal semen showed that sperm motility was significantly decreased (*P* ≤ *0.05*) in the Cd-treated group (51.44 ± 2.5) compared to the control group (93.7 ± 3.62). Co-administration of *Anacyclus* extract with cadmium significantly increased sperm motility (70.07 ± 3.42) compared to Cd-intoxicated rats (*P* ≤ *0.05*). In the *Anacyclus-*treated group, the percentage of sperm motility was 95.51 ± 3.85, which showed no significant difference compared to the control group (Fig. 2A).

Rats treated with 100 mg/kg *Anacyclus* extract had a sperm concentration of 79.11 ± 6.00, with no significant change compared to the control group (76.05 ± 5.22). The sperm concentration recorded in the cadmium-treated group (15.47 ± 1.94) was significantly lower (*P* ≤ *0.05*) than in the control group. The sperm concentration increased significantly (34.53 ± 3.88) when cadmium was combined with *Anacyclus* extract compared to the Cd-treated group (Fig. 2B).

### Sperm viability and morphology

The obtained results revealed that cadmium caused a significant decrease (*P* ≤ 0.05) in viability (47.53 ± 2.1) accompanied by a significant increase (*P* ≤ 0.05) in sperm abnormalities (26.09 ± 1.74) when compared to the control group (92.81 ± 3.27, 7.00 ± 0.48) respectively. In cadmium and *Anacyclus* treated group, the percentage of viable sperms (72.48 ± 3.27) was significantly increased (*P* ≤ 0.05), and the total sperm abnormalities (17.92 ± 0.82) was significantly decreased (*P* ≤ 0.05) compared to the Cd group while was still significantly higher than that of the control group (Fig. 2C). In addition, there were no significant differences of sperm viability (94.65 ± 3.69) and morphological abnormalities (6.84 ± 0.51) between *Anacyclus* extract treated group and the control group (Fig. 2 C, D).

### Sperm lipid peroxidation and antioxidant activities

**Fig. 3:**
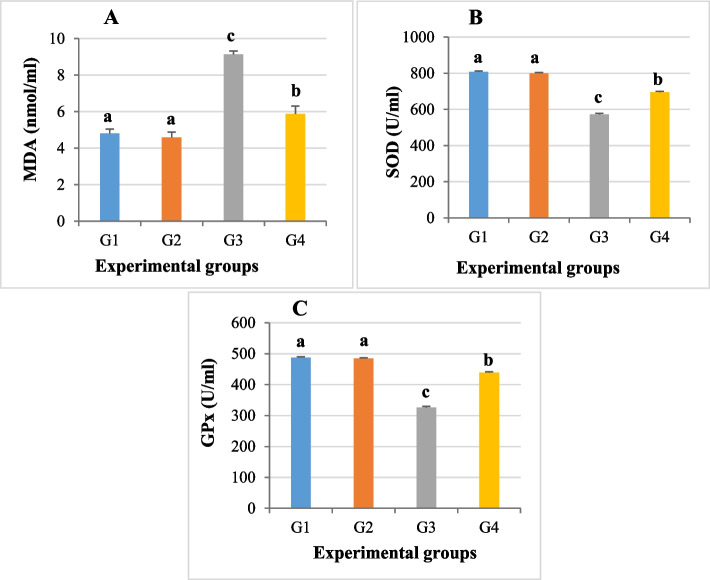
A histogram showing the effect of cadmium or/and *Anacyclus pyrethrum* extract administration for eight weeks sperm oxidative markers in all experimental groups. **A** The level of Malondialdehyde (MDA), (**B**) Superoxide dismutase (SOD), and **(C)** Glutathione peroxidase (GPx). G1: Control, G2: *Anacyclus* extract treated group, G3: Cadmium treated group, G4: Cadmium and *Anacyclus* extract treated group. Data is presented as mean ± standard error (S.E.). Different letters represent significant differences (*P* ≤ 0.05) between experimental groups

The MDA was significantly overproduced (*P* ≤ 0.05) in epididymal semen of Cd intoxicated rats (9.14 ± 0.18) compared to the control group (4.81 ± 0.24). A significant decrease (*P* ≤ 0.05) in the MDA level was observed upon the co-treatment of *Anacyclus* extract with Cd (5.87 ± 0.44) compared to the group that received cadmium alone. Moreover, there was no significant difference observed in the MDA level between the *Anacyclus* extract group (4.59 ± 0.29) and the control group (Fig. 3A).

Meanwhile, the concentrations of superoxide dismutase (SOD) and glutathione peroxidase (GPx) were significantly decreased (*P* ≤ 0.05) in the seminal fluid of the cadmium treated group (571.57 ± 6.84 for SOD and 326.42 ± 3.16 for GPx) compared to the control group (807.77 ± 4.51 and 487.14 ± 2.51, respectively). The concentrations of semen SOD and GPx showed a significant rise (*P* ≤ 0.05) in cadmium and *Anacyclus* treated group (694.71 ± 5.52 and 438.42 ± 3.18, respectively) compared to the semen of Cd intoxicated group. The enzyme levels in semen of rats exposed to 100 mg/kg *Anacyclus* extract were not considerably different from the control group with values of 798.71 ± 4.79 for SOD and 484.85 ± 2.29 for GPx (Fig. 3B, C).

### Reproductive hormones levels (FSH, LH and T)

**Fig. 4:**
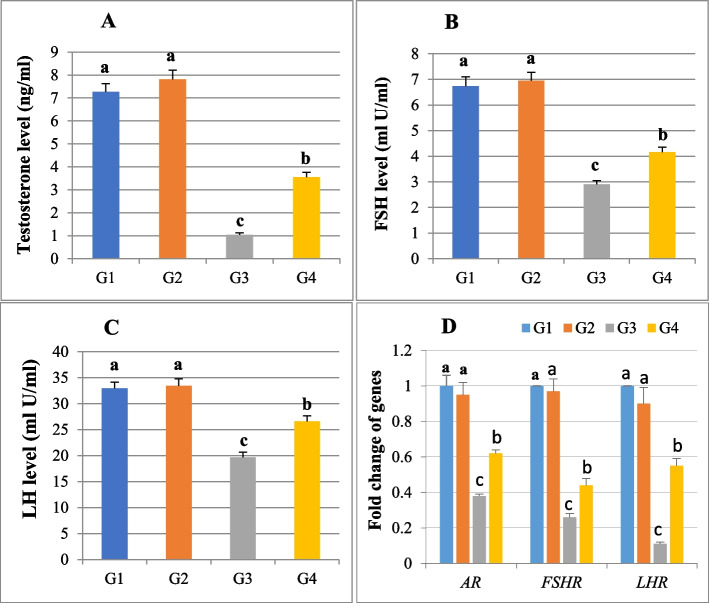
A histogram showing serum reproductive hormones levels and means of fold changes of hormone receptor genes in testicular tissue collected from all rats in the four groups. **A** Testosterone T, (**B**) Follicle-stimulating hormone FSH, (**C**) Luteinizing hormone LH, and (**D**) Follicle-stimulating hormone receptor (*FSHR*), luteinizing hormone receptor (*LHR*) and androgen receptor (*AR*). G1: Control, G2: *Anacyclus* extract treated group, G3: Cadmium treated group, G4: Cadmium and extract treated group. Data is presented as mean ± standard error (S.E.). Different letters represent significant differences (*P* ≤ 0.05) between experimental groups

The serum levels of FSH, LH, and T in rats exposed to 100 mg/kg of *Anacyclus* extract were 6.95 ± 0.32, 33.42 ± 1.36 and 7.81 ± 0.40, respectively, which did not significantly differ from that recorded for the control group (6.74 ± 0.36, 32.91 ± 1.22, and 7.27 ± 0.36). Cadmium- treated rats showed a significant drop in serum levels of the three reproductive hormones as compared to the control group (2.9 ± 0.15 for FSH, 19.64 ± 1.02 for LH, and 1.04 ± 0.09 for T). Meanwhile, the FSH, LH, and T levels were significantly increased in the Cd and *Anacyclus* treated group (4.16 ± 0.2, 4.16 ± 0.2, and 3.55 ± 0.21 respectively) compared to the Cd group levels (Fig. 4 A, B, C).

### Fold change of *FSHR*, *LHR* and *AR* genes

The relative gene expression of *FSHR***, ***LHR*, and *AR* in *Anacyclus* extract-treated rats was 0.97 ± 0.07, 0.90 ± 0.09, and 0.95 ± 0.07, respectively, showing no significant difference compared to the control group. In the cadmium-treated group, the mean of fold changes of *FSHR* (0.26 ± 0.02), *LHR* (0.11 ± 0.01), and *AR* (0.38 ± 0.01) were significantly decreased (*P* ≤ *0.05*) when compared to the control group. *Anacyclus* co-administration with cadmium resulted in a significant reduction in the mean of fold changes for *FSHR* (0.44 ± 0.04), *LHR* (0.55 ± 0.04), and *AR* (0.62 ± 0.02) compared to the control group (Fig. 4D).

### Assessment of sperm DNA fragmentation by comet assay (single cell gel electrophoresis, SCGE)

**Fig. 5:**
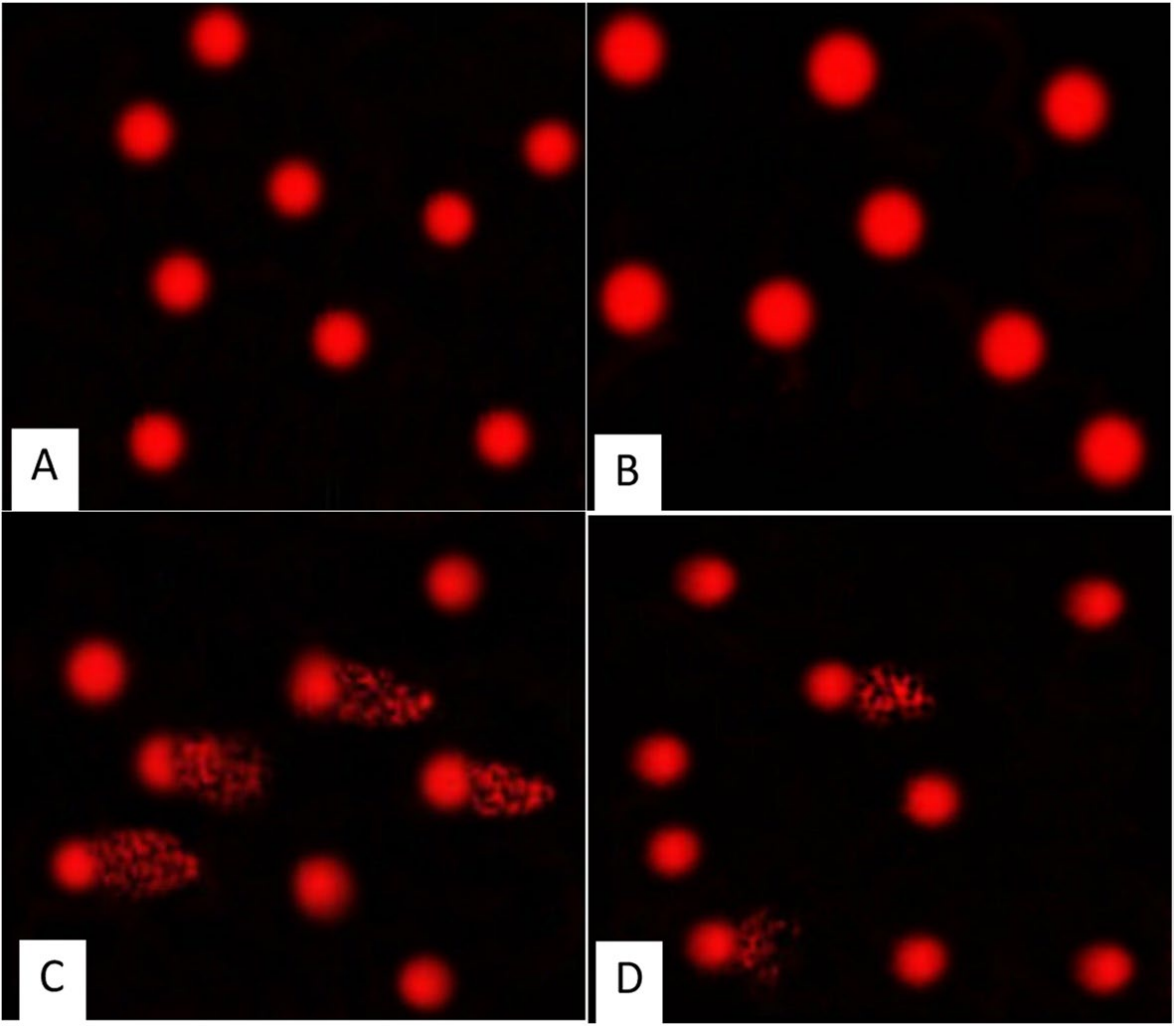
Photomicrographs showing the extent of DNA damage in sperms of rats in all experimental groups as detected by comet assay. **A** Control, (**B**) *Anacyclus pyrethrum* extract treated group, (**C**) Cadmium treated group, (**D**) Cadmium and *Anacyclus* extract treated group

The tail length and tail moment recorded in the *Anacyclus *extract treated group, were 2.10 ± 0.14 and 4.22 ± 0.16 respectively, showing insignificant difference compared to the control group (1.76 ± 0.13 and 2.97 ± 0.14). In the cadmium-treated rats, the tail length and tail moment (16.50 ± 0.72, 178.86 ± 8.29) were significantly increased (*P* ≤ 0.05) compared to the control group. The co-administration of *Anacyclus* with Cd significantly increased tail length and tail moment (7.65 ± 0.36, 42.99 ± 2.90) compared to the Cd treated group (Table 3, Fig. 5).

**Table 3:** Comet assay parameters obtained by image analysis for sperms

Group/parameter	Tailed %	Untailed %	Tails length µm	Tail DNA%	Tail moment
Control (G1)	2.5	97.5	1.76 ± 0.13 ^c^	1.69	2.97 ± 0.14 ^c^
*Anacyclus pyrethrum* (G2)	3.5	96.5	2.10 ± 0.14 ^c^	2.01	4.22 ± 0.16 ^c^
Cadmium (G3)	40	60	16.50 ± 0.72 ^a^	10.84	178.86 ± 8.29^a^
Cadmium and *Anacyclus* (G4)	22	78	7.65 ± 0.36 ^b^	5.62	42.99 ± 2.90^b^

Data is presented as mean ± standard error (S.E.). Means within the same column carrying different superscript letters are significantly different (*P* ≤ *0.05*)

### Apoptotic and pro-apoptotic gene fold changes (*Bax *and *Bcl2*)

**Fig. 6:**
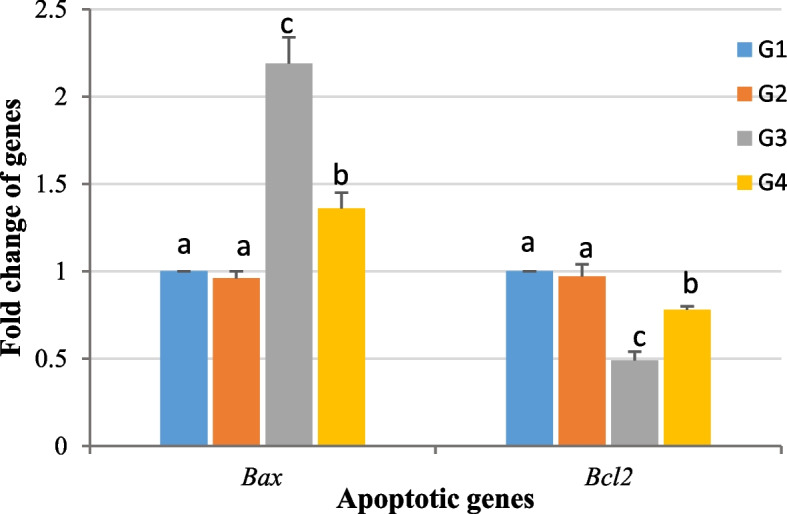
A histogram showing means of fold changes for Bcl-2 Associated X-protein (*Bax*), and B-cell lymphoma-2 (*Bcl-2*) genes in rats epididymal semen collected from all experimental groups. G1: Control, G2: *Anacyclus* extract treated group, G3: Cadmium treated group, G4: Cadmium and *Anacyclus* extract treated group. Data is presented as mean ± standard error (S.E.). Different letters represent significant differences (*P* ≤ 0.05) between experimental groups

The mean fold changes for *Bax* (0.96 ± 0.04) and* Bcl2* (0.97 ± 0.07) calculated for the semen of *Anacyclus-*treated group were not significantly changed compared to the control group. In the cadmium group, the fold mean change for *Bax* (2.19 ± 0.15) was significantly increased (*P* ≤ 0.05), while the fold change for *Bcl2* (0.49 ± 0.05) was significantly decreased when compared to the control group. In comparison to the cadmium treated group, the group co-administrated with *Anacyclus* and cadmium showed a significant decrease in the gene expression of *Bax* (1.36 ± 0.09) and a significant increase of *Bcl2* (0.78 ± 0.02) gene expression (Fig. 6).

## Discussion

Cadmium is widely used in different industrial applications producing high amounts of the heavy metal that is progressively accumulated owing to its long half-life. Cd toxicity might result in reproductive failure, infertility, DNA damage and cancer development [65].

A generally recognized mechanism of Cd reproductive toxicity is increasing the reactive oxygen species (ROS) which induces oxidative stress leading to compromised sperm quality, impaired spermatogenesis, DNA damage, inhibition of DNA repair system, disrupted hormonal functions, altered gene expression and apoptosis [66, 67]. Upon administration of Cd, we recorded a significant increase in MDA level in semen which might be due to the recorded significant decrease in the activity of the antioxidants (SOD and GPx) and the increased ROS production due to Cd toxicity [68, 69]. Research has shown that Cd preferentially binds to the sulfhydryl group (─SH)-containing molecules like SOD and GSH. The depletion of the ─SH group is considered as an indirect mechanism of Cd-induced oxidative stress [6, 14]. It was also reported that Cd might disrupt the expression of the antioxidant enzymes during the transcriptional stage [70]. The same observations were recorded in a previous work in caprine spermatogenic cells after Cd administration [6].

A decrease in MDA level and enhanced activities of SOD and GPx were observed in the semen of the group co-administered with *Anacyclus pyrethrum* and Cd. These results are congruent with the findings of El-Kholy et al. and Baslam et al. [42, 43, 47], who observed a reduction in MDA levels along with SOD, GPx elevation and suggested a therapeutic strategy of *Anacyclus pyrethrum* bioactive constituents by targeting oxidative stress in male rats’ testis and brain. Although the results of our study agree with previous studies where they proved the antioxidant activity of *Anacyclus pyrethrum*, none of them investigated the antioxidant activity of the plant extract in semen [32, 33].

We found that the administration of Cd resulted in a significant decrease in sperm concentration, motility, viability and altered sperm morphology. Although low levels of ROS are required for normal sperm concentration and functions, excessive ROS production might affect the semen quality by inducing peroxidation of polyunsaturated fatty acid (PUFA) and oxidative DNA strand breaks leading to impaired sperm concentration and functions [71, 72]. Several previous studies have reported a decrease in sperm concentration, motility and viability of mice, rats, and human as a result of exposure to cadmium [49, 54, 73, 74].

On the other hand, *Anacyclus pyrethrum* extract was able to significantly improve sperm parameters when administrated with Cd. In line with our findings, several previous studies proved that the extract of *Anacyclus pyrethrum* had the ability to improve the semen analysis parameters [35, 50, 75]. As detected by HPLC analysis, *Anacyclus* roots extract was shown to contain apigenin, rutin, ferulic acid, chlorogenic acid quercetin, myricetin, kaempferol and resveratrol. Previous research has shown that the antioxidant properties of these compounds improved sperm quality by lowering lipid peroxidation levels and increasing the antioxidant enzymes activities (SOD and GPx) [76–83].

In the current study, exposure to cadmium caused significant DNA damage as detected by the single-cell gel electrophoresis assay. Many recent studies demonstrated that sperm DNA integrity is a determining factor in normal fertilization and transmission of paternal genetic information. Consequently, examination of DNA integrity became a pivotal part in routine laboratory analysis to provide a full picture of semen functionality [84]. In a previous study, comet assay in epididymal sperms of adult male rats suffered from a significant DNA damage in all of the three Cd treated groups [85]. In our study we observed that administration of *Anacyclus pyrethrum* with Cd protects the sperm cells from DNA damage. The protective effect of *Anacyclus pyrethrum* might be attributed to its strong antioxidant effect which in turn prevents DNA oxidation and damage [33, 85]. The effective antioxidant properties of quercetin, rutin, gallic acid, rosmarinic acid, resveratrol, and apigenin might be the main reason for protecting sperms against oxidative DNA damage [83, 86–89]. Onuoha et al. [88] demonstrated the efficacy of combined administration of rutin, quercetin, and gallic acid in reducing cadmium-induced testicular DNA damage compared to their individual administration.

Cadmium acts as an endocrine disruptor and was proven to affect the hormones responsible for spermatogenesis and sperm development [72]. Our study revealed that cadmium administration significantly diminished the serum levels of follicle stimulating hormone (FSH), luteinizing hormone (LH) and testosterone (T). Similar results were found in previous work as they reported that cadmium significantly reduced the levels of LH, FSH and T in blood of rats [54, 90, 91]. They explained their findings by cadmium interference with the synthesis and regulation of the circulating levels of several hormones, including T, FSH, and LH [2, 91].

The co-administration of *Anacyclus pyrethrum* significantly elevated the blood circulating levels of LH, FSH and testosterone. In consistency with our results, previous studies found that the level of T, LH and FSH were significantly increased in the serum of albino rats after treatment with *Anacyclus pyrethrum* root extract [47, 49, 50]. This might be attributed to the ability of the extract to boost the function of Sertoli and Leydig cells as reported for the effect of walnut leaf extract in previous work [92]. According to the findings of Hasanein et al. [78], Osawe and Farombi [80] and Jaz et al. [81], Khafaji [82] the ameliorative effects of ferulic acid, quercetin, rutin, myricetin and kaempferol on Leydig and Sertoli cells may be involved in restoring serum reproductive hormones levels.

The present study showed that the relative mRNA expression levels of *FSHR*, *LHR*, *AR* genes were markedly downregulated in the Cd intoxicated animals which might affect the normal progression of spermatogenesis. Several previous studies indicated that intraperitoneal injection of low doses of Cd in rats downregulated relative mRNA expression of the same genes [93, 94]. On the other hand, we found that co-administration *Anacyclus pyrethrum* extract alleviated the effects of Cd on gene expression. A previous study showed the potential of kaempferol to upregulate the gene expression of LH and FSH receptors, thus improving the reproductive hormones production [82].

Oxidative stress-induced apoptosis in sperm is a critical regulator of fertility [95]. In our work, exposure to Cd pronouncedly altered mRNA expression of both *Bax* (pro-apoptotic gene) and *Bcl-2* (anti-apoptotic gene), where the expression of *Bax* was upregulated, and the expression of *Bcl-2* was downregulated indicating the induction of apoptosis. Long-term exposure to Cd^2+^ upregulates the expression of *p53* that binds with *Bax*, *Bcl-2*, and *Bcl-xl* to enhance pore formation in mitochondrial membrane. This is followed by releasing cyt-c to catalyze the activation of procaspase-3 to caspase-3 and causing apoptosis of the target cell [96]. Our observations were in line with the results recorded previously for Cd effect on the expression levels of *Bax* and *Bcl-2* genes in testis of mice and rats [6, 97]. *Anacyclus pyrethrum* extract was able to counter the apoptotic effects of Cd by downregulating the expression of Bax and upregulating the expression of *Bcl-2*. The presence of chlorogenic acid, apigenin, quercetin and hesperidin in the extract of *Anacyclus* roots may be attributed to the protection against testicular tissue apoptosis associated with oxidative stress [76, 79, 98, 99].

## Conclusion

The current work evaluated the effect of Cd toxicity on the semen quality. Besides significantly decreasing the semen analysis parameters (concentration, motility, viability, and morphology), Cd also significantly increased oxidative stress levels in the semen leading to a significant increase in DNA damage. The work also proved that Cd disrupted the circulating levels of LH, FSH, and T along with the modulation of mRNA expression levels of spermatogenic genes and *Bax*/*Bcl-2* ratio. This work and previous work showed that new semen parameters other than the traditional ones (like oxidative stress and gene toxicity) should be considered in routine semen analysis. The study also showed the ability of *Anacyclus pyrethrum* root extract to restore the semen qualities in Cd intoxicated rats.

## Supplementary Information

Supplementary Material 1.

## Data Availability

The datasets used and/or analysed during the current study are available from the corresponding author on reasonable request.

## Abbreviations

Cd: Cadmium
CdCl_2_: Cadmium chloride
SOD: Superoxide dismutase
GPx: Glutathione peroxidase
MDA: Malondialdehyde
T: Testosterone
FSH: Follicle stimulating hormone
LH: Luteinizing hormone
FSHR: Follicle stimulating hormone receptor
LHR: Luteinizing hormone receptor
AR: Androgen receptor
OS: Oxidative stress
ROS: Reactive oxygen species
HPLC: High Performance Liquid Chromatography
G1: Group (1) Control group
G2: Group (2) *Anacyclus pyrethrum* extract treated group
G3: Group (3) Cadmium treated group
G4: Group (4) Cadmium and *Anacyclus* extract treated group
EN: Eosin-nigrosin stain
PUFA: Polyunsaturated fatty acid
