# Supplementary material for: Anacyclus pyrethrum enhances fertility in cadmium-intoxicated male rats by improving sperm functions
Source: BMC Complement Med Ther. 2024 Nov 27;24:409. doi: 10.1186/s12906-024-04711-y (PMC11600599; doi:10.1186/s12906-024-04711-y)
Supplement: Supplementary file 1 — Supplementary Material 1. [file 12906_2024_4711_MOESM1_ESM.docx]

**The uncropped images of come assay**

**
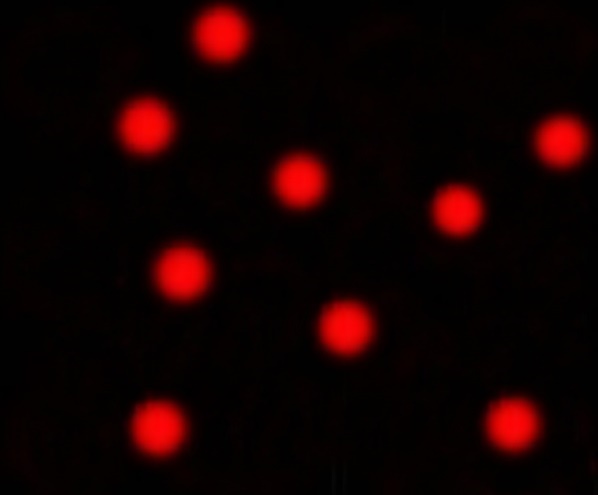

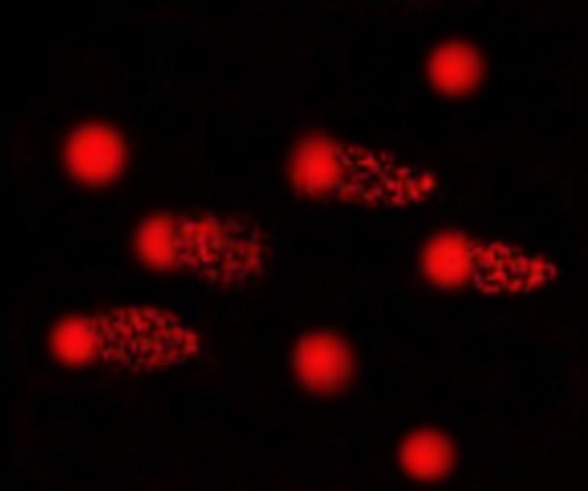

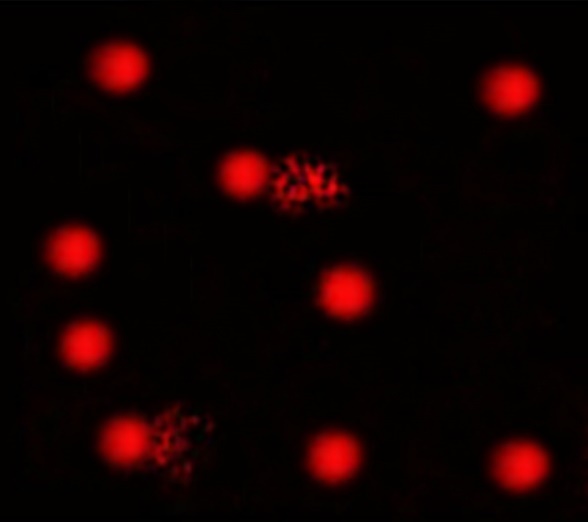

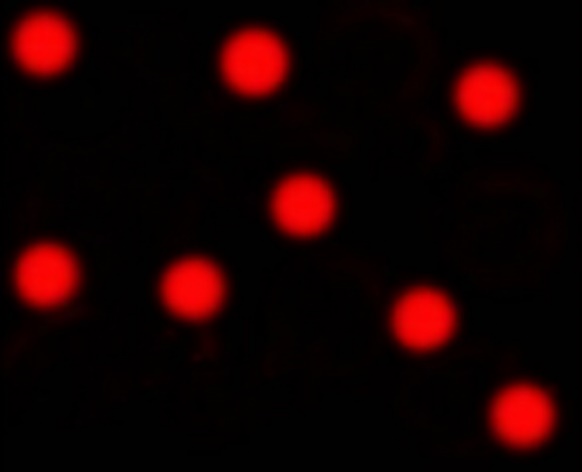
**

No other gel or western blot photos were included in the present work
